# Perspectives of aminoacylases in biocatalytic synthesis of *N*-acyl-amino acids surfactants

**DOI:** 10.1007/s00253-024-13328-7

**Published:** 2024-10-25

**Authors:** Gerrit Haeger, Jessika Wirges, Johannes Bongaerts, Ulrich Schörken, Petra Siegert

**Affiliations:** 1 Novo Nordisk, Novo Nordisk Park 1, 2760 Måløv, Denmark; 2 Institute of Nano- and Biotechnologies, Aachen University of Applied Sciences, Heinrich-Mussmannstr. 1, 52428 Jülich, Germany; 3 Faculty of Applied Natural Sciences, TH Köln University of Applied Sciences - Leverkusen Campus, 51379 Leverkusen, Germany

**Keywords:** *N*-acyl-amino acids, Aminoacylase, Biocatalysis, Biobased surfactants, Green chemistry

## Abstract

**Abstract:**

Many industrial processes are performed using harmful chemicals. The current technical synthesis of *N*-acyl-amino acids relies on acyl chlorides, which are typically obtained from phosgene chemistry. A greener alternative is the application of whole cells or enzymes to carry out synthesis in an environmentally friendly manner. Aminoacylases belong to the hydrolase family and the resolution of racemic mixtures of* N*-acetyl-amino acids is a well-known industrial process. Several new enzymes accepting long-chain fatty acids as substrates were discovered in recent years. This article reviews the synthetic potential of aminoacylases to produce biobased *N*-acyl-amino acid surfactants. The focus lays on a survey of the different types of aminoacylases available for synthesis and their reaction products. The enzymes are categorized according to their protein family classification and their biochemical characteristics including substrate spectra, reaction optima and process stability, both in hydrolysis and under process conditions suitable for synthesis. Finally, the benefits and future challenges of enzymatic *N*-acyl-amino acid synthesis with aminoacylases will be discussed.

**Key points:**

• *Enzymatic synthesis of N-acyl-amino acids, biobased surfactants by aminoacylases****.***

## Introduction to N-acyl-amino acids: properties, applications and biological occurrence

**Fig. 1:**
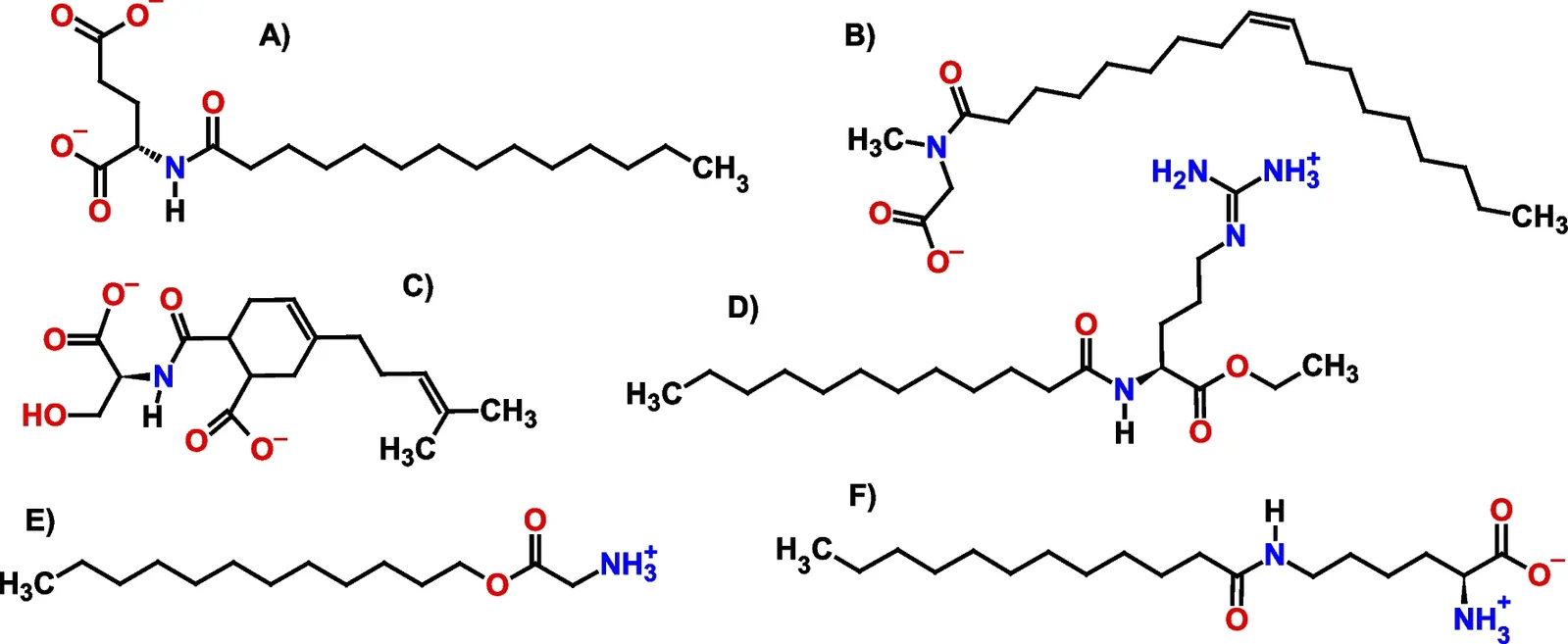
Exemplified structures of amino acid surfactants. **A** *N*-myristoyl-L-glutamate; **B** *N*-oleyl sarcosinate; **C** Diels–Alder adduct of myrcene, maleic acid and L-serine (Jolmes et al. 2024); **D** *N*-lauroyl-L-arginine ethyl ester (LAE); **E** O-lauryl glycinate; and **F** ε-*N*-lauroyl-L-lysine (Koreishi et al. 2009a)

More than 15 million tons of surfactants are produced annually, many of them still based on petrochemical raw materials. Their release into the environment necessitates the development of bio-based and environmentally friendly alternatives to replace conventional petrochemical-derived products such as difficult-to-degrade alkylbenzene sulfonates (Nagtode et al. 2023; Bhadani et al. 2020). Biogenic compounds suitable for the cost-efficient synthesis of biobased surfactants are manifold. Vegetable oil–derived fatty acids or fatty alcohols are typically used as hydrophobic tails, while the polar head groups can originate from a variety of biogenic compounds such as polyols, organic acids, sugars or amino acids (Nagtode et al. 2023; Foley et al. 2012). Glucose-derived non-ionic alkyl polyglycosides are the largest surfactant class by volume produced fully bio-based today (Schörken et al. 2017). In addition, a variety of anionic, amphoteric or cationic surfactants are accessible through the incorporation of amino acids and variation of both the amino acid and hydrophobic tail, which opens up a large structural space (Schörken et al. 2017; Pinheiro and Faustino 2017; Tripathy et al. 2018; Ananthapadmanabhan 2019). Due to their bi-functional structure, and in some cases a third functional side-chain group, amino acids possess multiple attachment points (Fig. 1). The carboxylic moiety offers esterification with fatty alcohols leading to cationic alkyl-amino acids, whereas the condensation of the amine group with fatty acids delivers anionic *N*-acyl-amino acids. Other hydrophobic tails may be attached to the amino acid, as was shown recently in the synthesis of Diels–Alder-type amino acid surfactants with terpenes (Jolmes et al. 2024). With cationic lysine or arginine, amphoteric *N*-acyl-amino acids are obtained and upon esterification of the carboxylic group; cationic acyl-amino acids like *N*-lauroyl-L-arginine ethyl ester (LAE) are accessible. Last but not least, functional side-chain groups like the *ε*-amine of lysine may be employed for hydrophobic group attachment (Koreishi et al. 2009a) or as linker for gemini-type amphiphile synthesis via dimerization (Morán et al. 2004). Today, straight-chain *N*-acyl-amino acids surfactants have the highest industrial impact and *N*-acyl glutamates, glycinates and non-proteinogenic sarcosinates and taurates are the major classes of amino acid surfactants utilized in personal care applications (Ananthapadmanabhan 2019). From these, *N*-acyl glutamates are probably the largest group of natural amino acid surfactants, originally developed by the Japanese company Ajinomoto Co., Inc. in the 1970s (Takehara et al. 1972b).

Acyl-amino acids are mild, possess little inflammatory potential and low toxicity, exhibit desirable foaming properties and are biodegradable. Overall, they have favorable properties compared to other anionic surfactants like sodium dodecyl sulfate (SDS) or sodium lauryl ether sulfate (SLES) (Tripathy et al. 2018; Ananthapadmanabhan 2019). Additionally, being valuable compounds in cosmetics, acyl-amino acids are remarkably skin-protective. The hygroscopic skin’s natural moisturizing factor comprise of amino acids and intercellular lipids in the stratum corneum and plays an important role in prevention of skin dehydration (Verdier-Sévrain and Bonté 2007). Enzymes that are able to hydrolyze acyl-amino acids were found in the human skin microbiome (Nagai and Matsuno 1964; Natsch et al. 2003). Acyl-amino acids can thus act as skin-protecting agents when decomposed after application. The surfactant properties depend on the length of the fatty acid and mild surfactants suitable for skin cleaning are based on lauric (C12) or myristic acid (C14). *N*-lauroyl-L-glutamic acid or *N*-oleoyl-L-glutamic acid are commercial products, the latter a more hydrophobic compound applied as an emulsifier in cosmetic formulations (Takehara et al. 1973). Hydrogen-bonding phenomena, pH-dependency of surface tension and self-assembly properties are positive characteristics of the glutamate-based acyl-amino acids (Ananthapadmanabhan 2019; Takehara et al. 1972b, 1972a; Takehara 1989).

The cationic surfactant LAE has strong antimicrobial properties and is therefore used as amphiphilic preservative in cosmetic and food applications. The cationic charge most probably damages cell membranes causing loss of membrane potential and membrane leakiness. Similarly, the coconut fatty acid derived arginine ethyl ester possesses antiviral and virucidal activities (Yamasaki et al. 2011). These cationic surfactants are applied in cosmetics as hair conditioners as they adsorb well onto hair and exhibit antistatic properties.

Acyl-amino acids also exhibit various physiological functions; for example, they show structural resemblance to the endocannabinoid *N*-arachidonoyl-ethanolamine (Battista et al. 2019). Several acyl-amino acids have been found in mammalian brains (Tan et al. 2009) and can act as signal molecule or interacting with G-protein-coupled receptors or other proteins. Furthermore, they were shown to stimulate mitochondrial oxidative metabolism through uncoupled respiration (Lin et al. 2018). Some short-chain acyl-amino acids or acylated dipeptides are neurotransmitters. *N*-acetyl-L-aspartate and *N*-acetyl-L-aspartyl-L-glutamate are the most abundant compounds found in the mammalian brain, respectively (Yan et al. 2003; Morland and Nordengen 2022). In addition, acyl-amino acids are found in many soil and marine microorganisms, and a large structural variability was detected in different species (Craig et al. 2011; Kubicki et al. 2019). Not all of their physiological functions are fully understood, though acting as signaling molecules and cellular messengers seems probable. Beyond that, microbial *N*-acyl tyrosines were shown to exhibit antibiotic properties against several bacteria and an oil degrading *Alcanivorax* strain produces *N*-acyl proline surfactants, which may be involved in alkane solubilization (Thies et al. 2016; Qiao and Shao 2010).

### Chemical and biological routes towards synthesis of N-acyl-L-amino acids

**Fig. 2:**
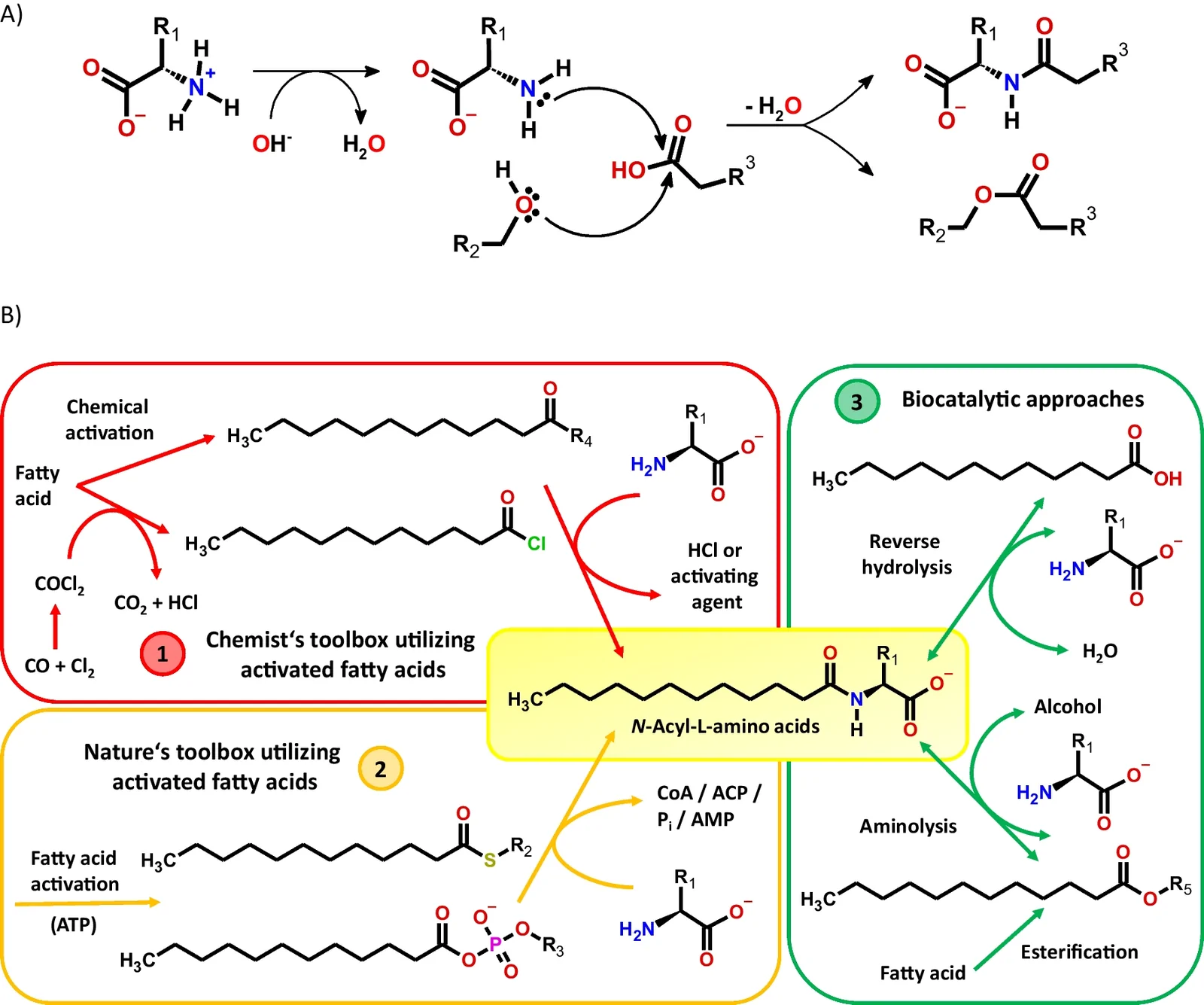
**A** Principal comparison of amide and ester synthesis utilizing amino acids or alcohols;** B** synthetic routes towards *N*-acyl-L-amino acids. Route 1: Chemical synthesis using coupling agents or chlorine chemistry; route 2: cellular systems employing activated fatty acids and route 3: in vitro biocatalytic approaches using either reverse hydrolysis or aminolysis; (*R*_1_ = amino acid side groups, *R*_2_ = CoA or ACP, *R*_3_ = H or adenylate, *R*_4_ = miscellaneous chemical activating groups and *R*_5_ = alcohols like methanol or glycerol, P_i_/AMP, inorganic phosphate/adenosine monophosphate). Processing details and responsible enzymes are described in the main text

Several routes towards *N*-acyl-L-amino acids are available including chemical synthesis and whole-cell biotransformation or in vitro biocatalysis (Fig. 2). Amide bond formation requires a nucleophilic attack of the *α*-amine. Comparing the reaction to ester synthesis, some differences become apparent. Though the amine is a better nucleophile than the alcohol hydroxy group, the synthesis of *N*-acyl-L-amino acids is more demanding. The zwitterionic nature of amino acids in aqueous environment necessitates proton abstraction from the ammonium, and a deprotonated or protected carboxylic group is needed as suitable electrophile (Fig. 2A). Additionally, the carboxylic acid group of the amino acid is a competing amidation site. To overcome these hurdles, chemists and nature evolved suitable strategies enabling selective synthesis.

#### Chemical synthesis of N-acyl-amino acids (Fig. 2B, route 1)

Until now, large-scale manufacturing is done chemically, though, regarding the “12 principles of green chemistry” (Anastas and Eghbali 2010), current processes suffer from several drawbacks including the utilization of toxic chemicals. A direct condensation of fatty acid and amino acid is not possible, and therefore an activation of the fatty acid is needed. In principle, this can be achieved with all coupling agents typically used in peptide synthesis (Valeur and Bradley 2009; Joullie and Lassen 2010). However, for cost reasons, these methods are only suitable for the lab-scale synthesis of pure compounds. The major industrial route proceeds via acyl chlorides, which are obtained from chlorination of fatty acids with phosgene (COCl_2_), an inherently toxic compound (Borak and Diller 2001), or in smaller scale with other chlorination agents like phosphorus trichloride (PCl_3_) or thionyl chloride (SOCl_2_). Phosgene itself is made from chlorine and carbon monoxide, and the fatty acid chlorination is usually conducted in the presence of the solvent dimethyl formamide to generate a reactive Vilsmeier complex for chlorination (Scott and Spedding 1968). The final condensation of the acyl chlorides is conducted under alkaline conditions in a Schotten-Baumann-type condensation. The reaction was adapted to amino acids by Ajinomoto and is run in water-solvent mixtures, whereby acetone is preferably used (Takehara et al. 1972b; Bordes et al. 2009). It is also possible to conduct the condensation of protein hydrolysates in water without organic solvents (Sander et al. 1997), though water acts as a competing nucleophile and hydrolysis of the acyl chlorides leads to increased fatty acid formation as byproduct. In the Schotten-Baumann reaction, the chlorine is released as hydrogen chloride and neutralized with base-generating sodium chloride in stoichiometric amounts. Additionally, nucleophilic-side groups like the *ε*-amine of lysine need protective group chemistry in case selective synthesis is desired. In summary, the need for hazardous chemicals, chlorine chemistry, use of organic solvents and stoichiometric generation of waste materials necessitates the development of more sustainable biocatalytic approaches towards *N*-acyl-L-amino acids.

#### Biological routes to N-acyl-L-amino acids (Fig. 2B, route 2)

The biological synthesis of *N*-acyl-L-amino acids resembles that of the chemical process in its need for fatty acid activation to shift the equilibrium towards condensation. This is achieved with activated acyl-phosphates, acyl-adenylates, acyl-coenzymeA (CoA) thioesters or with the help of acyl carrier proteins (ACP), which are formed under adenosine triphosphate (ATP) expenditure. Several enzymes including acyl-adenylating enzymes or *N*-acyl amino acid synthases (NAS) catalyze this condensation reaction. A summary of enzymes involved in a ATP-dependent *N*-acyl-amino acid synthesis was outlined in a recent concept study (Kua et al. 2024). In metagenome approaches, several microbial NASs with varying substrate selectivity were detected, and their expression in *E. coli* yielded the respective *N*-acyl-amino acids from the ACP-activated fatty acids (Brady et al. 2004; Brady and Clardy 2005). Though long-chain NASs seem to be widespread in bacteria (Craig et al. 2011; Kubicki et al. 2019), biotechnological utilization of these microorganisms or the heterologous expressed genes was not yet explored for a large-scale biosurfactant production. Meanwhile, some microbially produced lipopeptides like surfactin or daptomycin are commercially available in the meantime (Schörken et al. 2017), and in an interesting genetic engineering approach, the company Modular Genetics Inc. modified the surfactin synthesis pathway. Truncation of the nonribosomal peptide synthetase yielded a strain, which was able to synthesize *N*-acyl glutamates.

#### Biocatalytic synthesis of N-acyl-amino acid (Fig. 2B, route 3)

While the ATP-dependent *N*-acyl-amino acid synthesizing enzymes need a whole-cell biocatalysis for cofactor regeneration, the utilization of hydrolases in aminolysis or reverse hydrolysis reactions allows the direct use of the biocatalysts in vitro. Lipases and proteases were examined for this purpose. Screening the acylation potential of lipases with lysine and lysine-containing dipeptides as substrates revealed that the *ε*-amine of lysine was the preferred amidation site (Gardossi et al. 1991; Soo et al. 2003), which was confirmed in modeling studies (Dettori et al. 2018b). In general, the activity of lipases and proteases towards the *α*-amine was low, and better results were only achieved starting from fatty acid esters and/or the protected amino acids (Goujard et al. 2004). Thus, yields and conversion rates were judged to be too low for commercial implementation (Ansorge-Schumacher and Thum 2013). Some improvement was made by the utilization of silica-immobilized *Bacillus* protease and *Pseudomonas* lipase in polar acetone for the synthesis of *N*-acyl glycines. Synthesis yields of 40% were obtained with the protease in the reverse hydrolysis starting from non-protected glycine and lauric acid (Bernal et al. 2018). A further improvement in *N*-acyl glycine synthesis was recently achieved with a genetically modified *Rhizomucor* lipase. Glycerol as cosolvent led to an in situ formation of glycerol esters, followed by aminolysis of the ester to the acyl amino acid with up to 80% conversion (Kua et al. 2023). Still, the application of proteases and lipases suffers from a limited substrate scope, and the *α*-amine acylation of bulkier amino acids remains a challenge. The formation of amide bonds is valuable for the generation of pharmaceutical intermediates, and thus, selection and engineering of novel enzymes for amide bond is of high interest. Several new examples including, e.g. lipases (Zukic et al. 2024), promiscuous acyltransferases (Baumert et al. 2024), carboxylic acid reductases (CARs) or the combination of CARs and acyltransferase (Schnepel 2023). However, to the best of our knowledge, none of these enzymes was applied for the direct synthesis of *N*-acyl-amino acid starting from non-protected substrates up to now.

Hence, exploiting the synthetic potential of aminoacylases (EC 3.5.1.14) may be a more suitable approach. Aminoacylases have a long history in industrial biotechnology. Enantiomerically pure L-amino acids can be obtained from chemically synthesized racemic mixtures of acetyl-amino acids with these enzymes. Indeed, *Aspergillus oryzae* aminoacylase was one of the first enzymes with an industrial application and the first enzyme to be used in an immobilized form by Tanabe Seiyaku Co. Ltd. in 1969 (Chibata 1978). While chiral resolution by selective hydrolysis worked well with this enzyme, acylation yields in reverse hydrolysis trials were not satisfactory (Kimura et al. 1992). It needs to be emphasized that no driving force shifts the equilibrium towards N-acyl-amino acid synthesis in reverse hydrolysis trials. Thus, either a suitable micro-environment in the active site of the aminoacylases and/or the design of a suitable macro-environment is needed. Though aminoacylases and homologous peptidases are typically associated with *N*-acyl-L-amino acid hydrolysis, regulation of their in vivo cellular concentration by hydrolysis and synthesis was recently postulated for mammalian *N*-acyl amino acid synthase/hydrolase PM20D1 and a fatty acid amide hydrolase (Long et al. 2016; Kim et al. 2020). The first example of an enantioselective synthesis of *N*-acetyl-L-methionine was shown with the aminoacylase from porcine kidney (pAcy1) in 1994 (Yokoigawa et al. 1994). Several new aminoacylases were isolated in recent years, and synthesis of *N*-acyl-L-amino acids was proven with many of them (Table 1). Thus, their synthetic potential makes them promising enzymes for future technical applications.

**Table 1:** L-Aminoacylases described in literature

Organism	Name	MEROPS	Molecular mass (kDa)	X-mer	PDB	Recombinant	Synthesis	Ref
*Achromobacter pestifer*	ELA	−	−	−	−	−	−	(Chibata et al. 1964; Padayatty and van Kley 1967)
*Alcaligenes denitrificans*	−	−	40*	Dimer	−	−	−	(Yang et al. 1994)
*Arabidopsis thaliana*	ILR1, IAR3, ILL1-6	M20D	−	−	1XMB	*E. coli*	−	(LeClere et al. 2002)
*Aspergillus oryzae*	−	−	37	Dimer	−	−	−	(Gentzen et al. 1980)
*Bacillus subtilis*	AmhX	M20D	41.5	−	−	*E. coli*	−	(Kempf and Bremer 1996)
*Bacillus thermoglucosidius*	−	−	43*	Tetramer	−	−	−	(Cho et al. 1987)
*Burkholderia cepacian*	BcepM20D	M20D	42	Tetramer	−	*E. coli*	−	(Jamdar et al. 2015)
*Burkholderia lata*	Sgx9260c	M38	44.6	Octamer	3N2C	*E. coli*, IB	−	(Xiang et al. 2010)
*Burkholderia* sp.	−	M38	42*	Octamer	−	*E. coli* (low activity)	+ +	(Takakura and Asano 2019)
*Campylobacter jejuni*	HipO	M20D	42.6	−	−	*E. coli*	−	(Hani and Chan 1995)
*Caulobacter crescentus*	Cc2672	M38	45	−	3MTW	*E. coli*	−	(Xiang et al. 2010, 2009)
*Corynebacterium striatum*	CsAga	M20D	43.3	Dimer	6SLF	*E. coli*	−	(Natsch and Emter 2020; Natsch et al. 2003)
*Deinococcus radiodurans*	LAA	M20D	41.4	−	−	*E. coli*	−	(Hsu et al. 2006)
*Escherichia coli*	ArgE	M20A	42.3	Dimer	7RSF	*E. coli*	−	(Bourkaib 2020)
*Geobacillus stearothermophilus*	Ama	M20D	43	Tetramer	−	*E. coli*	−	(Sakanyan et al. 1993; Dion et al. 1995)
*Heliothis virescens*	L-ACY-1	M20A	50	Tetramer	−	*E. coli*	−	(Kuhns et al. 2012)
*Homo sapiens*	hAcy1	M20A	45	Dimer	1Q7L	*S. frugiperda*	−	(Lindner et al. 2003)
*Homo sapiens*	PM20D1	M20A	43	Dimer	−	293A cells	+	(Newman et al. 2007)
*Homo sapiens*	ASPA, Acy2	M14	43	Dimer	2I3C	*E. coli*	−	(Kaul et al. 1993; Herga et al. 2006)
*Lactococcus lactis* ssp*. cremoris*	Amd1	M20D	42	−	−	*L. lactis*	−	(Curley and van Sinderen 2000)
*Micrococcus agilis*	−	−	−	−	−	−	−	(Szwajcer et al. 1980)
*Mus musculus*	AcyIII	M14	35*	Dimer/tetramer	3NH4	HEK 293T	−	(Newman et al. 2007; Ryazantsev et al. 2007)
*Mycobacterium avium* Takeo	−	−	−	−	−	−	−	(Nagai 1961)
*Mycobacterium smegmatis*	−	−	40–48*	Monomer	−	−	−	(Matsuno and Nagai 1972; Nagai and Matsuno 1964)
*Mycolicibacterium smegmatis*	MsAA	M20A	48.6	Dimer	−	*E. coli*	+ +	(Haeger et al. 2023b)
*Paraburkholderia monticola*	PmAcy	M38	47.4	Dodeca-mer	−	*E. coli*	+ +	(Haeger et al. 2024)
*Paraburkholderia phytofirmans*	Sgx9260b	M38	45.3	Octamer	3MKV	*E. coli*, IB	−	(Xiang et al. 2010)
*Parkinsonia aculeata* L	−	−	−	−	−	−	−	(Lugay and Aronson 1969)
*Penicillium* sp.	−	−	−	−	−	−	−	(Murase et al. 1993)
*Pseudomonas diminuta*	Acy I Acy II	−	−	300/200*	−	−	−	(Fukuda et al. 1982)
*Pseudomonas* sp.	LpipACY	M38	45	Octamer	−	*E. coli*	−	(Hayashi et al. 2021)
*Pyrococcus furiosus*	−	M20D	42.0	Tetramer	−	*E. coli*, IB	−	(Story et al. 2001)
*Pyrococcus horikoshii*	PhoACY	M20D	43	Tetramer	−	*E. coli*	−	(Ishikawa et al. 2001; Tanimoto et al. 2008b)
*Staphylococcus aureus*	HmrA	M20D	43	Tetramer	3RAM	*E. coli*	−	(Botelho et al. 2011; Jamdar et al. 2015)
*Stenotrophomonas maltophilia*	−	M20	46	−	−	−	−	(Cao et al. 2020)
*Streptomyces ambofaciens*	SamAA	M20A	55	Monomer	−	*E. coli*, IB	+ +	(Bourkaib et al. 2020a; Bourkaib 2020)
*Streptomyces ambofaciens*	SamELA	M38	61	Monomer	−	*E. coli,* IB	+	(Bourkaib 2020; Dettori et al. 2018a)
*Streptomyces coelicolor*	ScELA	−	55.3	−	−	*S. lividans, E. coli, C. glutamicum*	+ +	(Takakura et al. 2009)
*Streptomyces griseus*	SgAA	M38	48.0	Dimer	−	*S. lividans*	+	(Haeger et al. 2023a)
*Streptomyces griseus*	SgELA	M38	56.9	−	−	*S. lividans*	−	(Haeger et al. 2023a)
*Streptomyces mobaraensis*	SmAA	M20A	55	Monomer	−	*S. lividans*	−	(Koreishi et al. 2009b)
*Streptomyces mobaraensis*	SmELA	M38	55	Monomer	−	*S. lividans, E. coli, C. glutamicum*	+ +	(Takakura et al. 2009; Koreishi et al. 2009a)
*Streptomyces mobaraensis*	−	−	100	Monomer	−	−	+	(Koreishi et al. 2005a)
*Streptomyces mobaraensis*	Penicillin V acylase	C59	19 + 61	Dimer	−	−	+	(Koreishi et al. 2006, 2007; Heckmann and Paradisi 2020)
*Sulfolobus solfataricus*	CPSso	M20D	43	Tetramer	4MMO	*E. coli*	−	(Sommaruga et al. 2014)
*Sus scrofa*	pAcy1	M20A	45.3	Dimer	−	*E. coli, S. frugiperda*	+	(Liu et al. 2006; Wardenga et al. 2008; Pittelkow et al. 1998)
*Thermococcus litoralis*	TliACY	M20D	43	Tetramer	−	*E. coli*	−	(Toogood et al. 2002; Parker et al. 2011)

“ELA” specificity of ε-lysine acylase. Sequences were subjected to MEROPS for classification (Rawlings et al. 2018). For published protein structure, the PDB-ID is annotated. Furthermore, if recombinant expression was shown the host is listed, the formation of inclusion bodies is indicated by “IB.” “ + ” indicates if synthesis of acyl-amino acids was shown in principle, remarkable conversions, or product concentrations, were designated with a double plus sign” +  + .” A single asterisk (*) indicates a molecular mass in kDa), determined by PAGE, native PAGE, or gel filtration. Adapted from (Haeger 2023)

### Aminoacylases belong to the metallopeptidase family

**Fig. 3:**
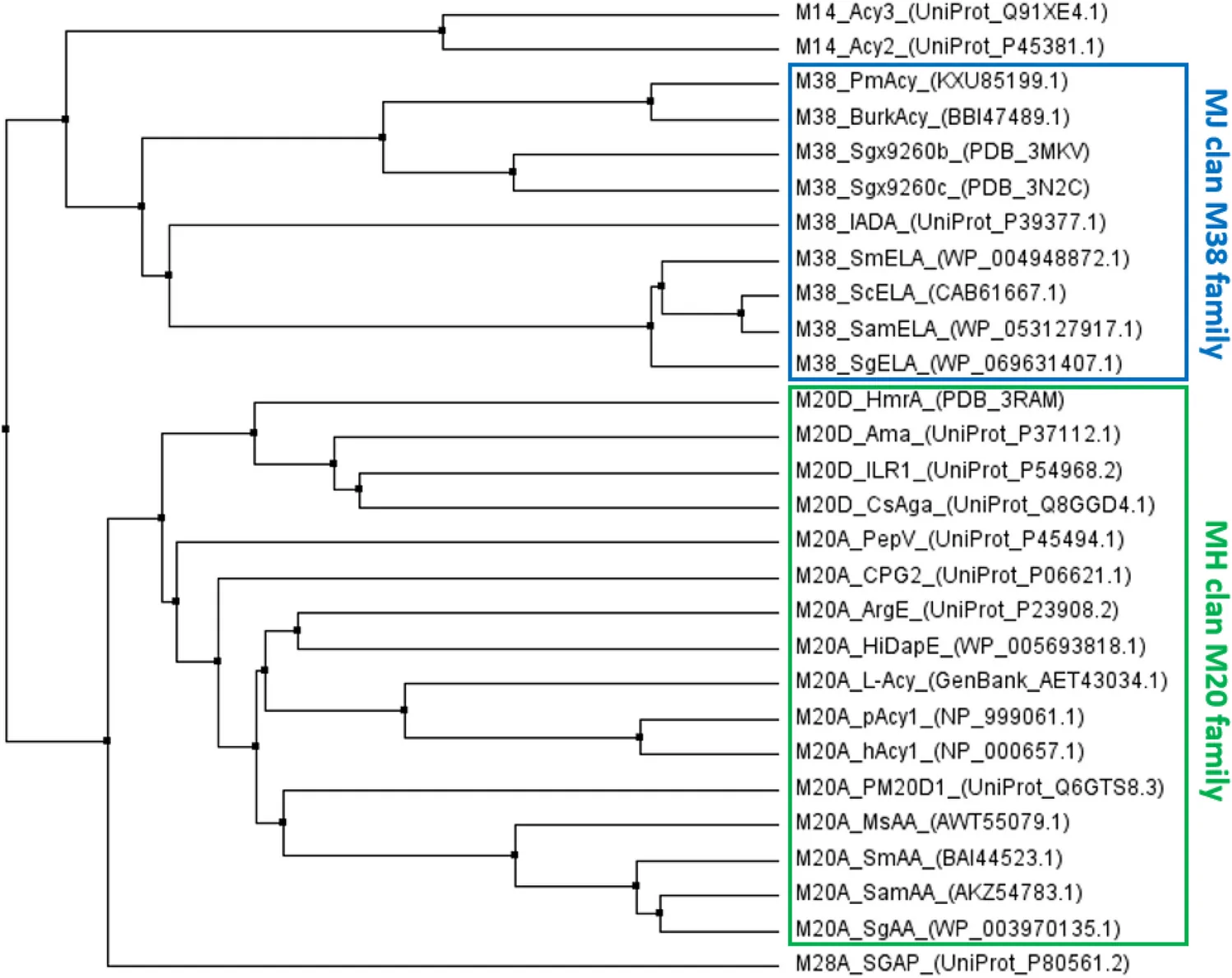
Phylogenetic tree of aminoacylases and relevant homologues. The alignment was generated with the Clustal Omega algorithm (Sievers et al. 2011) and displayed by average distance. To the enzymes acronyms their NCBI accession number, PDB- or UniProt identifiers are added (Haeger 2023). Acy3: murine aminoacylase-3 (UniProt: Q91XE4); Acy2, human aminoacylase-2 or aspartoacylase (UniProt: P45381); HmrA, peptidase from *Staphylococcus aureus* (PDB: 3RAM); Ama, aminoacylase from *Geobacillus stearothermophilus* (Uniprot P37112); ILR1, IAA-amino acid hydrolase from *Arabidopsis thaliana* (UniProt_P54968); CsAga, alpha-glutamine aminoacylase from *Corynebacterium striatum* Ax20 (UniProt_Q8GGD4); PepV, beta-Ala-Xaa dipeptidase from *Lactobacillus delbrueckii* (UniProt_P45494); CPG2, carboxypeptidase G2 from *Pseudomonas* sp. RS-16 (UniProt_P06621); ArgE, acetylornithine deacetylase from *E. coli* (UniProt*:* P23908); HiDapE, succinyl-diaminopimelate desuccinylase from *Haemophilus influenzae* (accession number: WP_005693818); L-ACY-1, aminoacylase 1 from *Heliothis virescens* (GenBank: AET43034); pAcy1, porcine aminoacylase-1 (accession number: NP_999061); hAcy1, human aminoacylase-1 (accession number: NP_000657); PM20D1, human N-fatty-acyl-amino acid hydrolase (UniProt: Q6GTS8); MsAA, aminoacylase from *Mycolicibacterium smegmatis* MKD8 (GenBank: AWT55079); SmAA, aminoacylase from *Streptomyces mobaraensis* (GenBank: BAI44523); SamAA, aminoacylase from *Streptomyces ambofaciens* ATCC 23877 (GenBank: AKZ54783); SgAA, aminoacylase from *Streptomyces griseus* (accession number: WP_003970135); PmAcy, aminoacylase from *Paraburkholderia monticola* (GenBank: KXU85199); BurkAcy, aminoacylase from *Burkholderia* sp. LP5_18B (GenBank: BBI47489); Sgx9260b, amidohydrolase (PDB: 3MKV); Sgx9260c, prolidase (PDB: 3N2C); IADA, isoaspartyl dipeptidase from *E. coli* (UniProt P39377) (Haley 1968); SmELA, epsilon-lysine acylase from *S. mobaraensis* (accession number: WP_004948872); SamELA, epsilon-lysine acylase from *S. ambofaciens* (accession number: WP_053127917); ScELA, aminoacylase from *S. coelicolor* A3 (GenBank: CAB61667.1); SgELA, epsilon-lysine acylase from *S. ambofaciens* (accession number: WP_069631407)

Aminoacylases hydrolyse C-N bonds that are not peptide bonds. Functionally and structurally, they share similarities with homologous metallopeptidases, and promiscuous activity may occur (Jamdar et al. 2015; Sakanyan et al. 1993; Ishikawa et al. 2001). Metallopeptidases cleave peptide bonds by an activated water molecule by a divalent metal cation, most often a zinc ion, but also cobalt, manganese, nickel or copper ions can be found. Depending on the number of metal ions in their active site, they can be classified into two groups: (I) contains one catalytic metal ion, while (II) requires two cocatalytic metal ions. The most common metal ligands are histidine, glutamic acid, aspartic acid, carboxylated lysine (Kcx) and cysteine. Glutamic acid acts as a general base (Rawlings and Barrett 2013). The sequences of almost all L-aminoacylases can be belong to the MEROPS M20 and M38 metallopeptidase families as non-peptidase homologues (Rawlings et al. 2018; Rawlings and Morton 2008). Only human aspartoacylase (ASPA or Acy 2), which cleaves acetyl-aspartate (Le Coq et al. 2006), mammalian aminoacylase-3 (Acy3), which deacetylates mercapturic acids, and *N*-acetyl-aromatic amino acids (Newman et al. 2007), belong to the M14 family. Penicillin V acylases (EC 3.5.1.11), also known to synthesize *N*-acyl-amino acids, belong to the MEROPS family C59 (Koreishi et al. 2006). A phylogenetic tree with aminoacylases and their homologues is shown in Fig. 3. Most of the aminoacylases investigated so far belong to the M20 metallopeptidase family. Despite overall low sequence similarity, their members have a highly conserved structure and conserved active site residues. Enzymes of this family are characterized by possessing two cocatalytic zinc ions, for which the binding sites are conserved. The family is further divided into subfamilies. Subfamily M20A contains dipeptidases, like peptidase V from *Lactobacillus delbrueckii* (PepV), carboxypeptidases and non-peptidase homologues, like acetylornithine deacetylase from *E. coli* (ArgE, EC 3.5.1.16) (Rawlings and Salvesen 2013; Boyen et al. 1992), *N*-succinyl-L,L-diaminopimelic acid desuccinylase from *Haemophilus influenzae* (HiDapE, EC 3.5.1.18) (Nocek et al. 2010) and several aminoacylases. Among them are the aminoacylases from porcine pAcy1 (Liu et al. 2006), the human hAcy1 PM20D1, (Lindner et al. 2003), SmAA from *S mobaraensis* IFO13819 (Koreishi et al. 2009b), SamAA from *S. ambofaciens* ATCC23877 (Bourkaib et al. 2020a), MsAA from *Mycolicibacterium smegmatis* MKD 8 (Haeger et al. 2023b) and SgAA from *Streptomyces griseus* DSM 40236 (Haeger et al. 2023a). Protein crystal structure has been solved from HiDapE (Nocek et al. 2010) and carboxypeptidase G2 (EC 3.4.17.11) from *Pseudomonas* sp. RS-16 (Rowsell et al. 1997). All residues essential for catalysis are conserved among these enzymes. The divalent metal ions, most often zinc, are coordinated by four or five ligands. It acts as a Lewis acid as the coordinated ion retains a positive charge (Bertini et al. 1985). The zinc site acts as a base by deprotonation, converting the bound water to a hydroxide ion (Rawlings and Barrett 2004). In M20A peptidases, the zinc ions are coordinated by two histidines and two glutamic acids. Aspartic acid is the bridging residue. The mechanism of hydrolysis of M20A enzymes has been studied in particular for HiDapE from *Haemophilus influenza* (Rawlings and Barrett 2013; Nocek et al. 2010, 2018; Auld 2013). The bound substrate interacts with one zinc ion with its amide carbonyl oxygen and disrupts the bridging water molecule. The catalytic glutamate acts as a general base by deprotonating the water molecule. The formed hydroxide ion attacks the carbonyl carbon of the substrate, forming a tetrahedral intermediate. The catalytic glutamic acid then donates a proton to the amide nitrogen, so the intermediate decomposes, and the products are released. The M20A aminoacylases contain the conserved amino acids of the metal-binding (H91, D123, E158, E185, H425) and the catalytic residues (D93, E157, H226, MsAA numbering) (Haeger et al. 2023b). The enzymes of the subfamily share a sequence identity of about 57–58% among the described aminoacylases (MsAA, SgAA, SamAA, SmAA; but 87.6% SmAA to SgAA) and only 26.2%, 25.6%, 21.5% and 23.1% to pAcy1, hAcy1, ArgE and HiDapE, respectively. MsAA, SgAA and pAcy were determined to be dimeric, and SmAA is described to be monomeric by native PAGE (Koreishi et al. 2009b) (Table 1). However, the histidine residues important for the function as a dimeric enzyme are conserved (Nocek et al. 2018). The peptidase subfamily M20D includes the peptidase with aminoacylase activity HmrA from *Staphylococcus aureus* (Botelho et al. 2011) as well as the aminoacylase from *Corynebacterium striatum* Ax20 (CsAga). Two cocatalytic zinc ions are bridged by a cysteine residue in CsAga, and the enzyme naturally occurs as a homodimer (Natsch 2015; Natsch and Emter 2020). The aminoacylase is specific for N_α_-acyl-glutamines, but promiscuous towards the acyl moiety, hydrolyzing lauroyl- or decanoyl-glutamine, benzoyl-glutamine, and branched chain 3-methyl-2-hexenoic-glutamine and 3-hydroxy-3-methylhexanoic-glutamine present in human axilla (Natsch et al. 2003). For the subfamilies M20B, C and F no enzyme with aminoacylase activity has been described yet. Also, several members of the M38 peptidase family belong to the amidohydrolase superfamily. The isoaspartyl-dipeptidase from *E. coli* has a homo-octamer structure (PDB 1ONW), and contains a binuclear metal active site for each subunit and catalyzes the hydrolysis of β-aspartyl dipeptides (Martí-Arbona et al. 2005a). One metal ion is coordinated by H68, H70, Kcx162 and D285, and the second metal ion is coordinated by Kcx162, H201 and H230. The lysine residue is carboxylated at the *ε*-amino group to form a carbamate, and bridges the two ions (Martí-Arbona et al. 2005a) whereas the residue Y137 contributes to the formation of an oxyanion hole and the stabilization of the transition state (Martí-Arbona et al. 2005b). Recently, further members of the M38 family have been described (Xiang et al. 2010). The aminoacylases from *Burkholderia* sp. LP5_18B (BurkAcy) (Takakura and Asano 2019) and *Paraburkholderia monticola* DSM 100849 (PmAcy) show sequence identities of 36.2 and 36.6% to the prolidase Sgx9260b from *Paraburkholderia phytofirmans* (PDB 3MKV), and 32.0 and 32.3% to Sgx9260c from *Burkholderia lata* (PDB 3FEQ). The prolidases hydrolyze various Xaa-Pro dipeptides, but also act on *N*-acyl-prolines like *N*-acetyl-L-proline or *N*-propionyl-L-proline. The sequence identity of PmAcy to BurkAcy is 85.5%. All metal-binding residues are conserved, as well as the oxyanion-hole forming histidine. PmAcy appears dodecameric (Table 1) in the native PAGE. The homologous enzymes, BurkAcy (Takehara et al. 1972b), L-pipecolic acid acylase from *Pseudomonas* sp. AK2 (LpipACY) (Hayashi et al. 2021), Sgx9260c and Sgx9260b (Xiang et al. 2009), or isoaspartyl dipeptidase (Martí-Arbona et al. 2005a), were found to be octameric (Xiang et al. 2010). Additionally, all *ε*-lysine aminoacylases (EC 3.5.1.17) known so far belong to the M38 peptidase family as well. The streptomycetal enzymes from *S. mobaraensis* (SmELA), *Streptomyces mobaraensis* IFO13819 (Takakura et al. 2009) and from *S. coelicolor* A3 (ScELA) (Takakura et al. 2009) are promising because of their high-level syntheses. All ELAs described so far show a sequence identity of > 80%. ScELA showed a sequence identity of 80.9% to SmELA and 93.6% to the enzyme from *S. ambofaciens* ATCC23877 (SamELA); albeit, sequence identities to PmAcy and BurkAcy are low with about 23%. The *ε*-lysine acylases also share the conserved residues with other members of the M38 peptidase family. However, the absence of the carboxylated lysine distinguishes the *ε*-lysine acylases from the other abovementioned M38 enzymes.

### Biocatalytic synthesis of N-acyl-amino acids

The acylation of amino acids with aminoacylases has been shown especially for the mammalian enzyme porcine, aminoacylase-1 (pAcy1) (Wardenga et al. 2010) and several streptomycetal aminoacylases. Namely, the *α*-aminoacylases from *S. ambofaciens* (SamAA) (Bourkaib et al. 2020a) and *S. griseus* DSM 40236 (SgAA) (Haeger et al. 2024) as well as the *ε*-lysine aminoacylases from *S. mobaraensis* (SmELA) (Koreishi et al. 2009a) and *S. coelicolor* (ScELA) (Takakura et al. 2009). Recently, syntheses with the *α*-aminoacylases from *Mycolicibacterium smegmatis* MKD 8 (MsAA) (Haeger et al. 2023b), as well as BurkAcy from *Burkholderia* sp. (Takakura and Asano 2019) and *Paraburkholderia monticola* (PmAcy) (Haeger et al. 2024) have been published (Table 2)*.* Different substrate specificities are observed for both the amino acid and the fatty acid moiety. A bias towards certain amino acids, e.g. preference of polar, charged, hydrophobic, small, or large side chains, occurs in most enzymes. Regarding the fatty acid specificity, the enzymes can be divided into short- or long-chain aminoacylases, and some of them additionally accept aromatic acyl residues. The human hAcy1 and the porcine pAcy1 are highly homologous. Both can hydrolyze diverse N_α_-acetyl-L-amino acids and exhibit a similar and broad substrate scope, with highest activity determined with norleucine (2S)-2-aminohexanoic acid), glutamate, leucine, methionine and glycine (Lindner et al. 2008b). Synthesis of various lauroyl-amino acids has been reported in a glycerol-water system for porcine pAcy1 (Wada et al. 2002). Highest conversion was observed for the synthesis of lauroyl-arginine (81.8%), probably due to its low solubility. Lauroyl-glutamic acid was produced with 44.4% conversion while other amino acids yielded between 0.9 and 35.1%. Only tyrosine and proline were not accepted at all. Concentrations were up to 0.5 M, or 1.0 M for glutamic acid, and lauric acid concentrations were 6.2 mM or 8.3 mM. Due to the small amount of fatty acid used for the acylations, final product concentrations remained low. With pAcy1, conversion for the synthesis of *N*-acetyl-methionine was only 18% in an aqueous system (Ferjancic-Biagini et al. 1997). In contrast to many other aminoacylases, glutamic acid could be acylated with pAcy1, albeit with low conversion rates of 1.5% and a 3.8-mM product from 1 M glutamic acid and 250 mM lauric acid methyl ester (Wardenga 2008). To improve pAcy1 through protein engineering, the variant D346A was generated, which improved the synthesis to hydrolysis ratio (Wardenga et al. 2010). However, an aggregation-prone-recombinant expression in *E. coli* and low-product concentration hamper the enzyme’s application in synthesis (Nocek et al. 2018). The human enzyme PM20D1 was also shown to have synthetic activity, especially for oleoyl-phenylalanine. However, the conversion was only 1.2% (Long et al. 2016). The enzyme SamAA isolated from *S. ambofaciens* ATCC 23877 catalyzes the acylation of amino acids with a broad substrate scope, and has been characterized mainly with 10-undecenoyl-phenylalanine. The reaction resulted in 5–23% conversion with non-polar as well as with positively charged amino acids and polar amino acids (Bourkaib et al. 2020a). Regarding the chain length of the fatty acid, the SamAA prefers middle-chain lengths like 10-undecenoic acid and lauric acid. A partly purified crude extract isolated from a wild-type strain was used for synthesis which was inactivated at 55°C and lost most of its synthetic activity after 16 h of incubation at 37°C (Bourkaib et al. 2020a, 2020b). Recently, the recombinantly expressed and purified *α*-aminoacylase MsAA from *Mycolicibacterium smegmatis* was characterized. As hydrolytic substrates, the enzyme prefers acetyl-amino acids, and activity with corresponding lauroyl-amino acids is lower. The enzyme has a bias for hydrophobic amino acids with a side chain, but substrates with bulkier hydrophobic or aromatic side chains are not hydrolyzed well. Some polar or charged acetyl-amino acids were hydrolyzed, like acetyl-aspartate, -glutamate, -arginine, -glutamine, -cysteine or threonine.

**Table 2:** Overview of the compounds synthesized by aminoacylases

Enzyme	Products	Amino acid [mM]	Fatty acid [mM]	Reaction conditions	Reference
***α*-aminoacylases**					
**hAcy1** (PM20D1)	*N*-oleoyl-phe, -leu, -val, -ile, -met, -gly, -ala, -gln, -lys, -ser	0.1	1.5	37°C, 1.5 h, pH not mentioned	(Long et al. 2016)
**pAcy1**	*N*-lauroyl-arg, -lys, -his	500	6.2	100 mM phosphate, pH 7.5, 25–33% (v/v) glycerol, 37°C, 144 h	(Wada et al. 2002)
	*N*-lauroyl-met			12.5% (v/v) 100 mM phosphate, pH 7.5, glycerol, 37°C, 24 h	
	*N*-lauroyl-asp, -gly, -ser, -thr, -cys, -asn, -val, -leu, -phe, -ile, -ala, -gln	500	8.3		
	*N*-lauroyl-glu	1000	250 (methyl ester)	50 mM Tris–HCl pH 8.5, 37°C	(Wardenga 2008)
**SamAA**	*N*-10-undecenoyl -lys, -arg, -leu, -gly, -ala, -val, -ile, -cys, -phe, -tyr, -trp, -ser, -thr, -gln, -asn, -his, -asp, -met	100	100	25 mM Tris–HCl, 50 mM NaCl, pH 8.0, 45°C, 3 days	(Bourkaib 2020)
	*N*-octanoyl -lys, -arg, -met, -leu, -phe				
	*N*-oleoyl-lys, -arg, -met, -leu, -phe				
**SmAA**	*N*-feruloyl-lys	3300	100	100 mM Tris–HCl, pH 7.2, 37°C, 4 days	(Koreishi et al. 2005a)
	*N*-feruloyl-phe, -met, -ser, -thr, -gln, -asn, -trp, -val, -leu, -ile, -gly, -ala, -glu, -asp, -his, -cys, -arg	500	10	100 mM Tris–HCl, pH 7.2, 70% glycerol, 37°C, 6 days	
SgAA*	*N*-lauroyl-met	100	100	100 mM Tris–HCl, pH 7.0, 40°C, 24 h	(Haeger et al. 2023a)
**MsAA**	*N*-lauroyl-met, -val, -ile, -leu, -ala, -phe	100	100	100 mM Tris–HCl, pH 7.0, 40°C, 3 days	(Haeger et al. 2023b)
**PmAcy**	*N*-lauroyl-his, -arg, -leu, -phe, -lys, -ile, -met, -val	200	100	50 mM Tris–HCl, pH 8.0, 50°C, 24 h	(Haeger et al. 2024)
	*N*-caproyl-phe				
	*N*-oleoyl-phe	200	100	50°C, 50 mM Tris–HCl, pH 8.0, 24 h + 10% EtOH (v/v)	
	*N*-stearoyl-phe				
	*N*-palmitoyl-phe				
**BurkAcy**	*N*-lauroyl-arg, -phe, -lys, -ala, -gly, -gln, -lys**, -ser, -val	200	100	100 mM Na-borate, pH 9.0, 60 h, 25°C	(Takakura and Asano 2019)
**Penicillin V acylase**	*N*-lauroyl-lys, -arg, -cys, -his, -ile, -val, -leu, -met, -gly, -ser, -asn, -glu, -thr	200	7.5	100 mM Tris–HCl, 0.5 mM CoCl_2_, pH 7.5, 2 days, 37°C, 78% glycerol (v/v)	(Koreishi et al. 2006, 2007; Heckmann and Paradisi 2020)
	*N*_ε_-lauroyl-lys				
***ε*-lysine aminoacylases**					
**ScELA**	*N*_ε_-lauroyl-lys	100	10	50 mM Tris–HCl, pH 7.0, 37°C	(Takakura et al. 2009)
**SmELA**	*N*_ε_-lauroyl-lys	500 500	50, 100, 250 10	100 mM Tris–HCl, pH 7.0, 37°C 100 mM Tris–HCl, pH 7.0, 45°C, 3 days	(Takakura et al. 2009; Koreishi et al. 2009a) (Koreishi et al. 2005b)

For the abbreviation of L-amino acids, the 3-letter code is used

*The syntheses by SgAA and MsAA were performed under initial, unoptimized conditions

**The authors did not distinguish between *α*/*ε* N-lauroyl-lysine

The designation of the enzymes is given in Table 1. Conversion/yields are discussed in the text

In summary, MsAA has a broad substrate spectrum regarding the amino acid moiety and prefers short acyl-chains in hydrolysis. A first, screening at unoptimized conditions for synthetic acylation activity towards all proteinogenic amino acids showed that several hydrophobic lauroyl-amino acids can be produced. Lauroyl-methionine was produced best with a 7.4-mM product from 100 mM lauric acid and methionine, respectively. Other *N*-acyl-amino acids produced were lauroyl-isoleucine, -leucine, -valine, alanine and -phenylalanine (Haeger et al. 2023b). Another homologous aminoacylase isolated from *S. mobaraensis* IFO 13819 (SmAA) has been cloned for expression in *S. lividans*. The enzyme has a broad acyl-chain specificity and can hydrolyze acyl-amino acids of different chain lengths. The highest hydrolytic activity was measured with acetyl-methionine, followed by acetyl-cysteine and -alanine, whereas acetyl-glutamic and -aspartic acid were not accepted. Activity against lauroyl-amino acids was lower than their acetyl-amino acids derivatives, similar to MsAA. With acyl-methionines of varying chain length from acetyl to palmitoyl residues, octanoyl-methionine was hydrolyzed best (Koreishi et al. 2009b). However, no synthesis with this enzyme could be shown.

Also, through recombinant expression in *S. lividans*, SgAA could be successfully produced. SgAA can hydrolyze a broad spectrum of acetyl-amino acids, and overall, the hydrolytic substrate scope was similar to that of MsAA. However, a 5.6-fold higher specific activity with N_α_-acetyl-arginine was observed for SgAA under the same assay conditions, and the enzyme was active with acetyl-proline, which MsAA is not. The substrates of SgAA with highest activity were acetyl-methionine, -alanine and -arginine. The activity with lauroyl-amino acids was rather low, which points out that SgAA is a short-chain-acyl aminoacylase. An initial screening for acylation of all proteinogenic amino acids with purified SgAA was performed. Under these non-optimized conditions, only lauroyl-methionine (4 mM) was produced (Haeger et al. 2023a), which is an interesting additive in cosmetic formulations with antioxidative properties (Wardenga et al. 2010)*.*

The biocatalytic potential of the aminoacylase PmAcy surpasses other processes for *α*-acylation of amino acids presented so far, especially with the enzyme being heterologously expressed in *E. coli* (Haeger et al. 2024; Siegert et al. 2023). A variety of amino acids could be acylated with conversions of 20–60%, including lauroyl-arginine (61%), -histidine (62%), -leucine (55%), -phenylalanine (48%), -lysine (30%), -isoleucine (14%), -methionine (12%), lauroyl and -valine (both 8%). The enzyme accepted various acyl donors, like caprylic (C8:0), lauric (C12:0), palmitic (C16:0), stearic (C18:0) and oleic acid (C18:1) with conversions of 49% to 75%, whereas the acids with longer acyl chains were converted best. Thus, the enzyme can be classified as a long-chain acyl-aminoacylase with a bias for hydrophobic amino acids. The substrate that was best accepted in hydrolysis was lauroyl-alanine, whereas acetyl-alanine was barely hydrolyzed. However, palmitoyl-alanine was hydrolyzed as well. The aminoacylase preferred lauroyl-glutamine over the shorter capryloyl-glutamine and the longer palmitoyl-glutamine. Among lauroyl-amino acids, favored substrates were -alanine, -methionine, -isoleucine, -valine and -glycine. Lauroyl-amino acids with aromatic side chain were also hydrolyzed, namely -phenylalanine, -tyrosine and rather poorly -tryptophan. Among the polar amino acids, only lauroyl-serine was hydrolyzed. No activity could be detected with lauroyl-aspartic acid, -glutamic acid and -cysteine (Haeger et al. 2024).

The homologous *α*-aminoacylase from *Burkholderia* sp. (Takakura and Asano 2019) also revealed a preference for hydrophobic amino acids, with lauroyl-alanine, -phenylalanine, -valine and -lysine being the preferred substrates in hydrolysis. Some lauroyl-amino acids with a polar side chain, like lauroyl-glutamine and lauroyl-serine, were hydrolyzed to a lesser extent. Lauroyl-glutamic acid and -aspartic acid were not accepted. BurkAcy synthesizes *N*-lauroyl-L-amino acids in high yields, e.g. *N*-lauroyl-arginine was produced with conversions of 89%. Other lauroyl-amino acids were formed efficiently as well, with highest conversion rates for lauroyl-phenylalanine (51%), -lysine (28%) and -valine (23%). Lauroyl-amino acids with negatively charged side chain were neither hydrolyzed nor synthesized. Extraordinary activity and stability at high temperatures and alkaline pH values were observed for PmAcy and BurkAcy. The pH optima for hydrolysis of both enzymes were pH 12.0, and they can thus be considered high-alkaline enzymes. Interestingly, the optimal pH for synthesis of acyl-amino acids differs vastly and lies at the slightly basic pH values of 8.0–9.0. The temperature optimum for hydrolytic activity for both enzymes was 70°C. BurkAcy was stable at 70°C for 60 min without loss of activity, and after 4 days at 70°C, 38% residual activity was determined (Takakura and Asano 2019). PmAcy also showed extraordinary stability even at pH 12.0 for 24 h. In summary, both enzymes are stable under typical process conditions, and together with the high conversion for acyl-amino acid synthesis are promising for industrial applications.

A few other long-chain aminoacylases have been described. Two enzymes were characterized from *Pseudomonas diminuta* (now *Brevundimonas diminuta)*. Aminoacylase I prefers long-chain acylglutamates, and highest activity was observed with pentadecenoyl-glutamic acid as a substrate, and no activity was measured with acetyl-glutamic acid (Fukuda et al. 1982). Long-chain aminoacylase II was isolated from the same organism and had a broader specificity and hydrolyzed lauroyl-glycine, -valine, -aspartic acid and -phenylalanine, and lauroyl-glutamic acid also (Shintani et al. 1984). Again, no activity with acetyl-glutamic acid was observed. Neither protein nor gene sequences were published for these aminoacylases. Two aminoacylases have also been described from *M. smegmatis* ATCC 607, but again no gene or protein is available. Aminoacylase I preferred long-chain acyl residues, while the other was a short-chain acyl aminoacylase. The long-chain aminoacylase hydrolyzes palmitoyl-aspartate, -valine, -phenylalanine, even arachidoyl-aspartate, but not acetyl-amino acids (Matsuno and Nagai 1972; Nagai and Matsuno 1964).

*N*_ε_-acyl-L-lysine is a detergent and disinfectant and also finds other industrial applications (Takakura et al. 2009). As these compounds are difficult to produce by the usually applied Schotten-Baumann reaction (Takakura et al. 2009), *ε*-lysine aminoacylases (ELA), which act on acyl-L-lysines in their *N*_ε_-position, are valuable enzymes. The *ε*-lysine aminoacylase produced by *S. mobaraensis* IFO13819 SmELA was found to be specific towards acylation of lysine at the *N*_ε_-position (Koreishi et al. 2009a). The conversion rates even reached 100% with decanoic acid, lauric acid and myristic acid, albeit with a 50-fold excess of lysine and a fatty acid concentration of only 10 mM (Koreishi et al. 2005b). Later on, the aminoacylase gene was cloned and heterologously expressed in *S. lividans*. Yields of 90–100% were observed with 500 mM lysine and 50 mM, 10–250 mM lauric acid after 6 h, 9 h, 37°C and 24 h, respectively (Koreishi et al. 2009a). The homologous *ε*-lysine acylase from *S. coelicolor* (ScELA) showed extraordinary synthetic activity towards N_ε_-lauroyl-lysine, with conversions reaching 100% (Takakura et al. 2009). Another homologue, SamELA from *S. ambofaciens*, was not applicable for N_ε_-acyl-lysine production, nor was SgELA, and the *ε*-lysine acylase from *S. griseus* DSM 40236, presumably because of its low stability (Haeger et al. 2023a). *ε*-Lysine aminoacylase activity has also been described for *Achromobacter pestifer*, but no synthesis has been shown (Chibata et al. 1964; Padayatty and van Kley 1967). Penicillin acylases are industrially used in synthesis of semi-synthetic antibiotics (Heckmann and Paradisi 2020), but were also capable of the acylation of amino acids. Penicillin V acylase from *S. mobaraensis* NBRC13422 was shown to acylate several amino acids, with free lauric acid (Koreishi et al. 2006) or by acyl transfer from methyl laurate (Koreishi et al. 2007). However, final product concentration remained low. The long-chain acyl-aminoacylase from *Penicillium* sp. B 001 is specific for acidic amino acids, with aspartic acid and only 25% of *N*-oleoyl-L-glu activity, and acyl moieties from C8 to C20 (*N*-arachinoyl-L-glu) are accepted with good activities (Nagao et al. 1992). But there is no information about the sequence of this enzyme available. Other examples for peptidases with *α*-aminoacylase activity but no demonstrated synthesis are carboxypeptidase from *Pyrococcus horikoshii* (Ishikawa et al. 2001), the aminoacylase from *Geobacillus stearothermophilus* (Sakanyan et al. 1993) or the peptidase HmrA from *S. aureus* (Jamdar et al. 2015) (Table 1).

### Heterologous expression of L-aminoacylases

Usually, recombinant protein expression starts with *E. coli* as production organism. However, recombinant production of aminoacylases with *E. coli* often turned out to be difficult. In particular, the formation of inclusion bodies presents a major obstacle (Takakura and Asano 2019; Bourkaib 2020; Wardenga et al. 2008). First experiments to express the human and porcine aminoacylase-1 pAcy1 in *E. coli* BL21(DE3) yielded in purified enzyme that hardly showed any activity (Pittelkow et al. 1998). In order to expresses additional tRNAs for rare eukaryotic codons, the *E. coli* strain BL21 Rosetta™(DE3) was developed. When this host was applied for pAcy1 expression, specific activities were obtained to compare the enzyme isolated from porcine kidney (Liu et al. 2006). With a codon optimized *pAcy1* gene and additionally co-expression of molecular chaperones, especially GroEL/S, expression of pAcy1 could be enhanced in *E. coli* BL21(DE3). However, the enzymes’ specific activity reached only half of the activity from pAcy1 isolated from porcine kidney (Wardenga et al. 2008). Only very low aminoacylase activity was obtained in the cell extract when the aminoacylase from *Burkholderia* sp. was expressed in *E. coli* BL21(DE3), and SDS-PAGE analysis did not show any visible overexpression (Takakura and Asano 2019). Further, aminoacylases successfully produced in recombinant *E. coli* were the aminoacylases from *Corynebacterium striatum* (Natsch et al. 2003), *Geobacillus stearothermophilus* (Sakanyan et al. 1993; Dion et al. 1995) the archaeon *Pyrococcus horikoshii* (Tanimoto et al. 2008a), PmAcy from *Paraburkholderia monticola* (Haeger et al. 2024) and MsAA from *Mycolicibacterium smegmatis* (Haeger et al. 2023b). Soluble expression of PmAcy with *E. coli* BL21(DE3) was obtained at 20°C by lactose-autoinduction and co-expressed chaperonine GroEL/S (Haeger et al. 2024). Similarly, MsAA production was increased with GroEL/S co-expression at reduced temperature. Further improvements were achieved using *E. coli* ArcticExpress (DE3) that constitutively expresses the cold-adapted chaperonins Cpn60/10 from *Oleispira antarctica* (Haeger et al. 2023b; Ferrer et al. 2003, 2004). In a patent filed by Ajinomoto Co., Inc. and Okayama University, *E. coli* JM109 was used to express the aminoacylases SmELA from *Streptomyces mobaraensis* and ScELA, a homologue from *S. coelicolor*, albeit with much lower yields than with *S. lividans* (Takakura et al. 2009). No soluble protein was obtained in attempts to produce the homologous aminoacylases SamAA and SamELA from *S. ambofaciens* in *E. coli* (Hani and Chan 1995), and the same result was obtained with the *S. griseus* enzymes SgAA and SgELA (Haeger et al. 2023a). Since *Streptomyces lividans* is the preferred expression host within the genus Streptomyces, *S. lividans* strains were considered as an alternative expression host to *E. coli* to produce streptomycetal aminoacylases (Koreishi et al. 2009a, 2009b; Haeger et al. 2023a). Unlike *E. coli*, Streptomyces do not tend to form inclusion bodies (Sevillano et al. 2016). Furthermore, *S. lividans* is known to exhibit low endogenous proteolytic activity (Berini et al. 2020). The strain *S. lividans* TK24 was used for heterologously production of the aminoacylases SmAA and SmELA from *S. mobaraensis* (Koreishi et al. 2009b, 2009a), whereas the aminoacylases SgAA and SgELA from *S. griseus* were produced with the strain *S. lividans* TK23 (Haeger et al. 2023a). In recent years, the gram-negative bacterium *Vibrio natriegens* turned out to be an attractive production host, not least due to its short-generation time of less than 10 min (Hoff et al. 2020; Hoffart et al. 2017). Besides the fast growth, the close genetic relationship to *E. coli* enables implementation of *V. natriegens* as an alternative production host, since many *E. coli* genetic elements as well as established molecular biology tools can easily be applied. For recombinant protein expression, the strain *V. natriegens* Vmax™ with a functional T7 expression system has been constructed (Weinstock et al. 2016). The aminoacylase MsAA from *M. smegmatis* was the first heterologously produced aminoaycylase by the strain Vmax™. Additionally, for the first time, chaperone co-expression in *V. natriegens* was reported, which significantly improved soluble aminoacylase expression (Haeger et al. 2023b). Instead of isolating human and porcine aminoacylase-1 from tissue, production of these enzymes with the Baculovirus expression system in *Spodoptera frugiperda* insect cells was applied (Pittelkow et al. 1998). The human aminoacylase PM20D1 was expressed with recombinant human 293A cell culture (Long et al. 2016).

## Conclusions and future areas of research

*N-*Acyl-amino acids are bio-based surfactants with a large structural variety and desirable chemical properties, including mildness and skin-friendliness, low inflammatory potential and good foaming ability. Additionally, they are environmentally benign and readily biodegradable. However, the current chemical processing requires chlorine chemistry, and hence, it is important to identify greener synthetic routes. Biocatalytic synthesis with aminoacylases is promising in this respect, and novel enzymes with synthetic potential were discovered in recent years. Nevertheless, to target technical implementation, more research is needed. *N*-acyl amino acid synthesis in reverse hydrolysis reactions requires reaction conditions, which enable an equilibrium shift towards amide bond synthesis. In lipase catalysis, several strategies were developed to shift the equilibrium towards ester synthesis. The main target is water activity (Petersson et al. 2007) and water removal may be achieved by, e.g. molecular sieves, the utilization of hydrophilic solvents like DES (deep eutectic solvents) (Kleiner and Schörken 2014) or the application of vacuum, which is established on industrial scale (Hills 2003). Setting up similar conditions for aminoacylase catalysis, biocatalysts with good process stability including high pH and temperature tolerance, broad substrate specificity and good activity in the presence of high substrate concentrations, in low-water environments or in the presence of solvents are needed. The recently investigated aminoacylases, PmAcy from *P. monticola* or the related enzyme from *Burkholderia* sp. possess several of these features and are candidates for future developments (Haeger et al. 2024; Takakura and Asano 2019). Reaction engineering including reactor design, enzyme immobilization and downstream processing must be considered as well. Reaction engineering for long-chain *N*-acyl-amino acid synthesis is a largely unexplored field and immobilization was only applied for the aminoacylases from *Streptomyces ambofaciens* so far (Dettori et al. 2018c; Bourkaib et al. 2021).

Still, the overall number of suitable aminoacylases for surfactant synthesis is low (Table 2). New aminoacylases with superior properties may be discovered for example from extremophile microorganisms. Currently, not many of the known aminoacylases exhibit sufficient synthetic activity with anionic aspartic and glutamic acid, and discovery of novel enzymes is needed here. In addition to sequence analysis, protein structure prediction can provide further insight into the properties of novel enzymes, but only few structures of L-aminoacylases have been solved and published, yet. No crystal structures of closely related aminoacylases (> 40% identity) to the ones with synthetic potential (Table 2) are available. Hence, more structural insight is needed. Molecular modeling and structure prediction with, e.g. AlphaFold (Jumper et al. 2021), and may help identifying amino acids responsible for substrate specificity or synthetic activity. Many research works cover the optimization of the synthetic potential of lipases and acyltransferases (Subileau et al. 2017; Müller et al. 2020), and engineering of lipases for *N*-acyl-glycine synthesis was recently shown (Kua et al. 2023). In comparison, until now only few examples for protein engineering of aminoacylases can be found. Mainly, variants were generated to understand functions of certain residues, e.g. investigating metal-binding, catalysis and substrate binding (Liu et al. 2006; Lindner et al. 2005, 2008a, 2003). Only two examples target the optimization of aminoacylases for synthetic purposes. Variants of pig kidney aminoacylase pAcy1 were successfully designed to improve the synthesis to hydrolysis ratio (Wardenga et al. 2010), and *Thermococcus litoralis* TliACY variants with increased thermostability were generated (Parker et al. 2011). So far, no enzyme engineering was applied for the tailoring of aminoacylase substrate specificities, and therefore, enzyme engineering holds promise for future aminoacylase optimization. Although some progress concerning the recombinant expression of aminoacylases has been made recently, further development for a cost-efficient production is necessary. Finally, promiscuous reactions have been described for many enzymes including lipases and also some aminoacylases (Hult and Berglund 2007). With aminoacylase from *Aspergillus melleus*, the enantioselective acylation of amines with vinyl acetate was proven (Youshko et al. 2001). Reactions with non-natural substrates like amines or alcohols may lead to novel bio-based surfactants, though the acylation with aliphatic fatty acids remains to be shown.
